# Factors Influencing Cecal Intubation Time during Retrograde Approach Single-Balloon Enteroscopy

**DOI:** 10.1155/2014/212307

**Published:** 2014-11-20

**Authors:** Tzu-Ming Ou, Peng-Jen Chen, Yu-Lueng Shih, Meng-Ting Wang, Hsin-Hung Huang, Wei-Kuo Chang, Tsai-Yuan Hsieh, Tien-Yu Huang

**Affiliations:** ^1^Division of Gastroenterology, Department of Internal Medicine, Tri-Service General Hospital, National Defense Medical Center, No. 325, Sec. 2, Cheng-Gong Road, Neihu, Taipei 114, Taiwan; ^2^Department of Internal Medicine, Kaohsiung Armed Forces General Hospital, No. 2, Zhongzheng 1st Road, Lingya, Kaohsiung 802, Taiwan; ^3^School of Pharmacy, National Defense Medical Center, No. 161, Sec. 6, Minquan E. Road, Neihu, Taipei 114, Taiwan

## Abstract

*Background and Aim*. The predisposing factors for prolonged cecal intubation time (CIT) during colonoscopy have been well identified. However, the factors influencing CIT during retrograde SBE have not been addressed. The aim of this study was to determine the factors influencing CIT during retrograde SBE. *Methods*. We investigated patients who underwent retrograde SBE at a medical center from January 2011 to March 2014. The medical charts and SBE reports were reviewed. The patients' characteristics and procedure-associated data were recorded. These data were analyzed with univariate analysis as well as multivariate logistic regression analysis to identify the possible predisposing factors. *Results*. We enrolled 66 patients into this study. The median CIT was 17.4 minutes. With univariate analysis, there was no statistical difference in age, sex, BMI, or history of abdominal surgery, except for bowel preparation (*P* = 0.021). Multivariate logistic regression analysis showed that inadequate bowel preparation (odds ratio 30.2, 95% confidence interval 4.63–196.54; *P* < 0.001) was the independent predisposing factors for prolonged CIT during retrograde SBE. *Conclusions*. For experienced endoscopist, inadequate bowel preparation was the independent predisposing factor for prolonged CIT during retrograde SBE.

## 1. Introduction

Single-balloon enteroscopy (SBE) is a safe and effective technique for the diagnosis and treatment of small bowel disease. Indications include obscure gastrointestinal bleeding, unexplained abdominal pain, chronic diarrhea, suspected inflammatory bowel disease, or suspected small intestinal tumor. The procedure can be performed via the antegrade or retrograde route. Based on the results of previous studies and clinical experience, retrograde SBE is more difficult than antegrade SBE because of the factors such as redundancy of the colon, looping of the scope, inadequate bowel preparation, and the time-consuming process of passing through the enteroscope through the colon [1–3]. The instruments for SBE consist of an enteroscope and an overtube with an inflatable balloon on its tip. The enteroscope is thinner than the standard scope used for colonoscopy and has no stiffening device, which makes controlling more difficult than that for colonoscopy. In addition, the skills needed for intubating for SBE are different from those for colonoscopy. The coordination of the enteroscope and the overtube is time-consuming during insertion. Difficulty in passing the enteroscope through the whole colon can increase the total procedure time and can result in the need for more sedation, external abdominal compression, and change of position, as well as causing discomfort [4–8]. Therefore, identifying factors that influence the ease of insertion of SBE in the colon is important for improving the quality of SBE.

Several studies have identified the predisposing factors for prolonged cecal intubation time (CIT) during colonoscopy, but to our best knowledge, none have addressed CIT during retrograde SBE. Previous studies have found older age [4–8], female gender [4, 5, 7–10], lower body mass index (BMI) [4, 5, 8, 10], smaller waist circumference [5], poor bowel preparation [4–7, 10], poor quality sedation [11], and prior hysterectomy [12] as predisposing factors for prolonged CIT during colonoscopy. However, it is not known whether they are predisposing factors for prolonged CIT during retrograde SBE. The aim of this retrospective study was to determine whether these factors influence CIT during retrograde SBE.

## 2. Materials and Methods

From a review of case notes, we identified all the SBE cases performed at Tri-Service General Hospital from January 2011 to March 2014. Patients who had a history of total or partial colectomy were excluded. The indications for retrograde SBE included small intestinal tumor, obscure gastrointestinal bleeding, unexplained abdominal pain, chronic diarrhea, and suspected inflammatory bowel disease. The characteristics of the cases, including age, sex, BMI, and history of abdominal surgery, were recorded (BMI was calculated as body weight divided by height squared [kg/m^2^]). Bowel preparation was performed with polyethylene glycol solution or sodium phosphate.

The bowel preparation level was classified as excellent, good, fair, or poor in accordance with the recommendation of American College of Gastroenterology and the American Society for Gastrointestinal Endoscopy [13]. We defined the excellent and good levels as adequate bowel preparation; meanwhile, we defined the fair and poor levels as inadequate bowel preparation. All retrograde SBE procedures were performed with an Olympus single-balloon enteroscope (SIF-Q260; Olympus Optical, Tokyo, Japan) by an experienced endoscopist (more than 30 cases of retrograde SBE experiences) [1]. Carbon dioxide was insufflated during the procedure. Before SBE, hyoscine-*N*-butylbromide, midazolam, and meperidine were administered to the patients on demand as a bolus. There was a cap attached at the tip of enteroscope to improve the visual field and mucosal hooking during the procedure. The SBE procedure was performed according to the instructions for the single-balloon enteroscope system from Olympus. Patient lay in the left lateral position at the beginning and changed to supine position by the demand of operator. The balloon of splinting tube was inflated followed by withdrawal of the splinting tube for shortening the colon while difficult to advance the scope in the colon lumen. Finding the appendiceal orifice and ileocecal valve indicated that the cecum had been reached. Total procedure time was defined as the time from SBE insertion until its removal from the patient. The CIT and total procedure time were recorded. Prolonged CIT was defined as more than the mean CIT in our study. Any findings from the enteroscopy and intervention were also recorded. Serum amylase level on the second day after the procedure was recorded, as was follow-up if any adverse event developed. This study was approved by the Institutional Review Board of Tri-Service General Hospital.

*Statistical Analysis*. All data were presented as the mean ± standard deviation for continuous variables, or the number (percentage) for categorical variables. Statistical analysis was performed using PASW statistics software, version 18 (IBM Co., Somers, NY, USA). Continuous variables were compared using Student's *t*-test, and categorical variables were compared using chi-square or Fisher's exact tests. All reported *P* values were two-tailed, and *P* < 0.05 was considered statistically significant. The general characteristics of the cases were analyzed. Univariate analysis was performed to determine potential predictive factors for prolonged CIT. Multivariate logistic regression analysis was used to identify which factor(s) were independent predisposing factors for prolonged CIT.

## 3. Results

### 3.1. Cases Characteristics

From January 2011 to March 2014, 141 patients underwent SBE, of which 74 underwent retrograde SBE. Of the 74 patients, 8 were excluded due to a history of total or partial colectomy. We therefore enrolled 66 patients into our study. The mean age of population was 56.4 (range 21–87) years (Table 1). There were 34 men and 32 women. Forty-four patients underwent retrograde SBE owing to obscure gastrointestinal bleeding, 9 due to small intestinal tumor, 7 due to unexplained abdominal pain, 4 due to suspected inflammatory bowel disease, and 2 due to chronic diarrhea. Twenty patients underwent abdominal surgery, of which 4 had pelvic surgery.

### 3.2. SBE Characteristics

Bowel preparation was excellent in 23 patients, good in 27, fair in 16, and poor in 0 (Table 2). The mean CIT was 17.4 ± 7.0 (range 3–33) min. The CIT was longer than mean CIT in 27 patients. The total procedure time was 77.2 ± 26.1 (range 29–175) min. Twenty-six patients had negative findings, 17 had ulcerative lesions, 9 had tumor lesions, 5 had angiodysplasias, 3 had diverticular lesions, 2 had nonspecific mucosal erosions, 1 had one stricture lesion, and 3 revealed polyps. These lesions were all detected in the small intestine. Overall, there were positive findings in 60.6% (40/66) patients. Thirty-three patients did not receive any intervention, 27 underwent biopsy, 3 underwent argon plasma coagulation, and 3 underwent polypectomy. The overall intervention rate was 50.0% (33/66). After polypectomy, 1 patient developed delayed bleeding and 1 patient developed perforation. The postintervention adverse event rate was 6.1%. No adverse events were found in those who did not receive any intervention. Hyperamylasemia was found in 5 patients. However, there was no case of acute pancreatitis. Univariate analysis showed that, with the exception of inadequate bowel preparation (*P* = 0.021), there was no statistical difference in CIT when comparing age, sex, BMI, history of abdominal surgery, and sequence of the procedure (Table 3). Furthermore, multivariate logistic regression analysis showed that inadequate bowel preparation (odds ratio 30.2, 95% confidence interval 4.63–196.54; *P* < 0.001) was the independent predisposing factor for prolonged CIT, rather than older age, female gender, lower BMI, prior abdominal surgery, and earlier procedure of all cases (Table 4).

## 4. Discussion

Because balloon-assisted enteroscopy is a time-consuming procedure, any manipulation to shorten the procedure time has the clinical impact for improving the quality of balloon-assisted enteroscopy. Our study disclosed that inadequate bowel preparation is the independent factor to influence the CIT of retrograde single-balloon enteroscopy, not other possible factors (e.g., age, BMI, gender, or previous abdominal surgery). It is interesting to get this result because these other possible factors are influencing factors for CIT of colonoscopy. Although we did not carry out the head-to-head comparative study, it could give us some hints that we could not use those influencing factors of CIT in colonoscopy to predict the CIT of retrograde single-balloon enteroscopy except the bowel preparation. Several previous studies identified older age, female gender, lower BMI, smaller waist circumference, poor bowel preparation, poor quality sedation, and history of hysterectomy as predisposing factors for prolonged CIT in colonoscopy. However, most of these predisposing factors did not affect the CIT of retrograde SBE in our study. These influencing factors might be overcome by balloon-assisted procedure except inadequate bowel preparation. In previous studies, balloon-assisted colonoscopy could resolve the problem of difficult/incomplete colonoscopy [14]. Inadequate bowel preparation directly influences vision during the insertion stage and results in a greater time needed for suction and water irrigation. In addition, stool located between the endoscope and the overtube increases friction and thus worsens coordination, which also contributes to prolonged CIT. The preparation of colon may not be overcome using an overtube. It takes a great deal of time to remove stool and to visualize the path forward. It could make sense from the knowledge of operation process of retrograde balloon-assisted enteroscopy and the application of balloon-assisted colonoscopy for the cases with difficult colonoscopy.

Prolonged CIT means that endoscopists spend additional or more time inserting the enteroscope from the anus to the cecum. Prolonged CIT during retrograde balloon-assisted enteroscopy might cause more discomfort of patient, requirement for more sedation, and more pain control and increase the load of endoscopist and assistant. From the clinical practice and studies, retrograde approach enteroscopy is more difficult than antegrade approach enteroscopy. The major reason is that it is not easy to pass through the scope from anus to cecum. We need to spend more time to overcome the difficulty in operating the balloon-assisted enteroscope. Although the procedure time of retrograde SBE mostly depends on the working process in the small bowel, which is variable and uncontrollable, CIT can directly influence the total procedure time. What we can do about improving the quality of retrograde SBE is to shorten the CIT, which leads to a shorter total procedure time and reduces unfavorable events as mentioned above. Therefore, we focused on the CIT rather than the time after intubating cecum. In addition to the CIT, successful terminal ileum intubation is another challenge while operating the scope in the retrograde procedure; it may take longer time to intubate the terminal ileum sometimes. This depends on many factors, including looping in the right colon, retroflexion in the cecum, sharp angle between the ascending colon and the terminal ileum, inadequate position of balloon inflation in the colon, and inability to intubate the terminal ileum deeply. However, the influencing factors of terminal ileum intubation in retrograde SBE need to be further addressed. Ng et al. [15] demonstrated that cap-assisted colonoscopy shortens the CIT compared with that after standard colonoscopy. Although it is not standard practice to attach a cap to the tip of enteroscope, the cap provides better visual fields for procedure performance. In our cases, the cap was attached to our enteroscope for every procedure; therefore, the influence of the cap can be discounted. However, whether the cap attachment could shorten the CIT during retrograde SBE needs further investigation. In addition, a previous study reported that an adequate learning curve for the procedure of retrograde SBE is 20 to 30 cases [1]. In our study, these procedures were performed by an experienced endoscopist. In addition, the univariate and multivariate logistic regression analyses both showed that the sequential procedures of retrograde SBE did not influence CIT in our study.

The results of our study disclose some practical implications. First, the use of the balloon-assisted equipment in retrograde SBE might overcome some factors that predispose to prolonged CIT in colonoscopy; it could be also confirmed by the studies of applying balloon-assisted colonoscopy in difficult or incomplete colonoscopy [14]. Kaltenbach et al. [16] reported that double-balloon enteroscopy facilitates cecal intubation after incomplete colonoscopy. Therefore, in addition to the balloon-assisted colonoscopy, single-balloon enteroscopy could apply to the case with difficult or incomplete colonoscopy. Second, we found that inadequate bowel preparation was the predisposing factor for prolonged CIT during retrograde SBE. Therefore, adequate bowel preparation before retrograde SBE could make the procedure easier and shorten the procedure time. It should be noticed to the patient, patient family, and medical personnel before the procedure.

The limitations of our study were its retrospective nature and small sample size. Further prospective studies with a larger sample size are needed to confirm the findings of the current study. In conclusion, we found that inadequate bowel preparation was the independent predisposing factor for prolonged CIT during retrograde SBE. Adequate bowel preparation is extremely important for shortening the procedure time and improving the quality of retrograde SBE.

## Figures and Tables

**Table 1:** Clinical characteristics of the patients enrolled for retrograde SBE (*n* = 66).

Characteristics	*n* (%)
Age (years)^a^	56.4 ± 19.8 (range 21–87)
Sex (male/female)	34/32
Body mass index (kg/m^2^)^a^	22.7 ± 3.0 (range 15.7–33.3)
Indications	
Obscure gastrointestinal bleeding	44 (66.7)
Small intestinal tumor	9 (13.6)
Unexplained abdominal pain	7 (10.6)
Suspected inflammatory bowel disease	4 (6.1)
Chronic diarrhea	2 (3.0)
History of surgery	20
Upper abdomen	4
Gastrectomy	1
Cholecystectomy	3
Lower abdomen	12
Small bowel resection	3
Appendectomy	7
Herniorrhaphy	2
Hysterectomy	4

^a^Values are mean ± standard deviation.

**Table 2:** The endoscopic findings of retrograde SBE (*n* = 66).

Finding	*n* (%)
Bowel preparation	
Excellent	23 (34.8)
Good	27 (40.9)
Fair	16 (24.2)
Poor	0 (0)
CIT (min)^a^	17.4 ± 7.0 (range 3–33)
Total procedure time (min)^a^	77.2 ± 26.1 (range 29–175)
Endoscopic diagnosis	
Negative	26 (39.4)
Ulcerative lesion	17 (25.8)
Tumor	9 (13.6)
Angiodysplasia	5 (7.6)
Diverticulum	3 (4.5)
Nonspecific mucosal erosion	2 (3.0)
Stricture	1 (1.5)
Polyp	3 (4.5)
Intervention	
None	33 (50.0)
Biopsy	27 (40.9)
Argon plasma coagulation	3 (4.5)
Polypectomy	3 (4.5)

^a^Values are mean ± standard deviation.

**Table 3:** Univariate analysis of predisposing factors for prolonged CIT.

Factor	*n*	CIT^a^ (min)	*P* value
Age (years)			0.258
<60	34	16.1 ± 7.6
≥60	32	18.8 ± 6.1
Sex			0.522
Female	32	18.8 ± 6.5
Male	34	16.0 ± 7.3
BMI (kg/m^2^)			0.599
<23	32	16.3 ± 6.8
≥23	34	18.3 ± 7.1
Bowel preparation			0.021
Adequate	50	15.2 ± 6.1
Inadequate	16	24.2 ± 4.9
Prior abdominal surgery			0.214
No abdominal surgery	46	16.0 ± 7.2
Abdominal surgery	20	20.6 ± 5.5
Sequence of the procedure^b^			0.300
Earlier cases (1–33)	33	16.8 ± 6.8
Later cases (34–66)	33	17.9 ± 7.3

^a^Values are mean ± standard deviation.

^b^All cases were divided into two groups by the sequence of procedure.

**Table 4:** Multivariate logistic regression analysis to determine predisposing factors for prolonged CIT.

Factors	Odds ratio (95% CI)	*P* value
Age ≥ 60 (years)	0.95 (0.22–4.01)	0.940
Female	2.94 (0.72–12.12)	0.135
BMI < 23 (kg/m^2^)	1.51 (0.41–5.57)	0.540
Inadequate bowel preparation	30.2 (4.63–196.54)	<0.001
Prior abdominal surgery	3.35 (0.81–13.80)	0.095
Earlier procedure of all cases^a^	2.01 (0.52–7.84)	0.315

CI, confidence interval, BMI, body mass index.

^a^Defined as first half of all cases by sequence.
